# New Highlights of Resveratrol: A Review of Properties against Ocular Diseases

**DOI:** 10.3390/ijms22031295

**Published:** 2021-01-28

**Authors:** Dominique Delmas, Clarisse Cornebise, Flavie Courtaut, Jianbo Xiao, Virginie Aires

**Affiliations:** 1 Université de Bourgogne Franche-Comté, F-21000 Dijon, France; clarisse.cornebise@gmail.com (C.C.); flavie.courtaut@gmail.com (F.C.); virginie.aires02@u-bourgogne.fr (V.A.); 2 INSERM Research Center U1231, Cancer and Adaptive Immune Response Team, Bioactive Molecules and Health Research Group, F-21000 Dijon, France; 3 Centre Anticancéreux Georges François Leclerc, F-21000 Dijon, France; 4 Nutrition and Bromatology Group, Department of Analytical Chemistry and Food Science, Faculty of Food Science and Technology, University of Vigo-Ourense Campus, E-32004 Ourense, Spain; jianboxiao@yahoo.com; 5 College of Food Science and Technology, Guangdong Ocean University, Zhanjiang 524088, China; 6 International Research Center for Food Nutrition and Safety, Jiangsu University, Zhenjiang 212013, China

**Keywords:** resveratrol, polyphenols, nutraceutical, ocular diseases, eyes, AMD, angiogenesis, diabetic retinopathy, cataract

## Abstract

Eye diseases are currently a major public health concern due to the growing number of cases resulting from both an aging of populations and exogenous factors linked to our lifestyles. Thus, many treatments including surgical pharmacological approaches have emerged, and special attention has been paid to prevention, where diet plays a preponderant role. Recently, potential antioxidants such as resveratrol have received much attention as potential tools against various ocular diseases. In this review, we focus on the mechanisms of resveratrol against ocular diseases, in particular age-related macular degeneration, glaucoma, cataract, diabetic retinopathy, and vitreoretinopathy. We analyze, in relation to the different steps of each disease, the resveratrol properties at multiple levels, such as cellular and molecular signaling as well as physiological effects. We show and discuss the relationship to reactive oxygen species, the regulation of inflammatory process, and how resveratrol can prevent ocular diseases through a potential epigenetic action by the activation of sirtuin-1. Lastly, various new forms of resveratrol delivery are emerging at the same time as some clinical trials are raising more questions about the future of resveratrol as a potential tool for prevention or in therapeutic strategies against ocular diseases. More preclinical studies are required to provide further insights into RSV’s potential adjuvant activity.

## 1. Introduction

**Figure 1:**
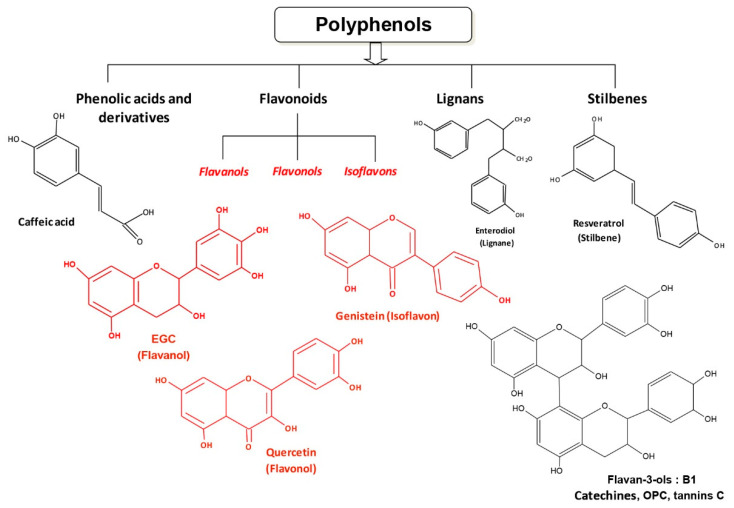
Chemical structures of polyphenols. Polyphenols that are ubiquitous in plants can be divided into four classes: phenolic acids such as caffeic acid, flavonoids including quercetin and genistein, the lignans, and the stilbenes, to which resveratrol belongs.

The number of visually impaired people of all ages is estimated to be 285 million worldwide, of whom 39 million are blind [1]. The leading causes of blindness and low vision are primarily age-related eye diseases such as age-related macular degeneration (AMD), cataract, diabetic retinopathy (DR), and glaucoma. The molecular mechanisms seem to be involved in many common steps, such as early oxidative stress, an inflammatory-associated process, and often over-angiogenesis. Visual impairment is a major global health issue, partly due to an aging world population, and the search for preventive or therapeutic strategies is therefore an important issue. Thus, of the dietary microcomponents that can participate in these strategies against ocular diseases, polyphenols could be good candidates (Figure 1). Indeed, various studies have shown that polyphenols may protect against numerous diseases (i.e., vascular diseases, cancers, and associated inflammatory disorders) [2,3,4]. These phytomolecules have cellular targets similar to those of the new drugs developed by pharmaceutical companies. Indeed, more than 1600 patents are currently reported concerning flavonoids and 3000 patents concerning polyphenols. Pleiotropic pharmaceutical activities are claimed in fields such as cancer, inflammatory arthritis, eye diseases, and many other domains. One of the best known is the polyphenol resveratrol, which is a *trans*-3,4′,5-trihydroxystilbene (*Trans*-RSV) (Figure 1) and appears to be of great interest in the prevention of these pathologies. Resveratrol (RSV) presents a myriad of beneficial health effects and acts at multiple levels such as cellular signaling, enzymatic pathways, apoptosis, and gene expression to prevent or fight coronary heart damage, cancer, and degenerative diseases in in vitro and in vivo studies [5,6,7,8]. Due to its multiple biological properties, RSV could help fight the main molecular events of ocular pathologies through its antioxidant power, as well as its anti-inflammatory and anti-angiogenic properties. In this review, we focus only on RSV with whom we have been working for more than 20 years on the elucidation of biological properties and also because it is one of the only polyphenols so that there are both very many robust studies on the molecular mechanisms on several ocular pathologies but also some clinical studies. Thus, this present review concentrates on the current knowledge of the mechanism of RSV on some of the main ocular disorders such as AMD, glaucoma, cataract, and DR. We describe cellular and molecular actions of RSV, as well as its potential epigenetic action in ocular disease prevention. Furthermore, a number of clinical trials have been conducted to test the efficacy of RSV in some cases of AMD. Lastly, we discuss the potential use of RSV and its new formulations in association with other therapeutic drugs.

## 2. The Origins of Resveratrol

RSV is a secondary metabolite produced in a small number of plant species. It was first isolated in 1940 from the root of *Veratrum grandiflorum*, which is a common flower of the Eurasian grasslands [9]. The root of the word “resveratrol” is a combination of the Latin prefix *Res*, meaning “which comes from”, *veratr*, from the plant “Veratrum”, and the suffix *ol*, indicating that it contains “alcohol” chemical groups*. Veratrum grandiflorum* has been reported to synthesize RSV and analogues. It is interesting to note that root powder of *Veratrum album* has long been used at medium altitude in Northern Europe, Asia, and Japan to treat rheumatism and nervous diseases. However, *Veratrum album* contains potent toxic alkaloids: the protoveratrines A & B. The RSV precursor is phenylalanine and the key cellular enzyme is stilbene synthase, which initiates the synthesis pathway toward RSV instead of toward flavonoids through chalcone synthase [10]. Therefore, RSV can be classified either as a stilbene or as a polyphenol (Figure 1).

Firstly, a significant amount is found in the leaves when the plant is damaged by chemical treatments [11]; secondly, the roots and rhizomes are relatively rich in RSV and, as such, were used as a crude preparation to treat hypertension in the East (Table 1) [12].

One of the richest sources of RSV is Japanese knotweed (*barnbuo japonaise*) *Polygonum cuspidatum*, root extract. These roots play an important role in ancient Chinese and Japanese natural medicine [13,14]. Its presence has been reported in many trees including eucalyptus [15,16], the spruce [17], and tropical trees like *Bauhinia racemosa* [18]. RSV has been identified in a small number of flowering plants; only two species of hellebore, *Veratrum grandiflorum* and *Veratrum formosanum*, are able to synthesize this compound. RSV is also present in *Pterolobium hexapetallum*, a legume [19]. Cotyledons such as peanut *Arachis hypogaea* synthesize a set of stilbene—phytoalexins including RSV with concentrations significantly increased in response to infection, injury, and ultra-violet (UV) irradiation [20,21,22,23,24].

The importance of RSV in plant biology lies in its ability to inhibit the growth of fungal infection, a property that has allowed it to be included in the class of antibiotic plants: phytoalexins. Due to its function as a phytoalexin and its role as a marker of infection by various pathogens, this interest in RSV mainly concerns vines (*Vitaceae*). The first report describing the structure and presence of RSV in grapes and its induction by infection with a fungus such as *Botrytis cinerea* [25,26,27] showed that this hydroxystilbene can exist in two forms: *trans*-RSV, the most organic form actively blocking the development of *Botrytis cinerea*, and *cis*-RSV obtained by the action of light on the *trans*-form. Most grape varieties contain varying quantities of this natural fungicide molecule, with grape skin containing about 50–100 µg of RSV per gram. It follows that during maceration, which takes place in the process of red wine vinification, RSV is dissolved in the hydro-alcoholic medium and then found in abundance in red wine, i.e., up to 20 mg/L [28]. Furthermore, RSV can be converted into viniferin by the action of vacuolar peroxidases [27,29]. Methyl-transferase and glucosyl-transferase activities have been identified to explain the biosynthesis of pterostilbene and RSV glucosides (including piceid and RSV-3-O-β-d-glucoside) [30,31,32].

**Figure 2:**
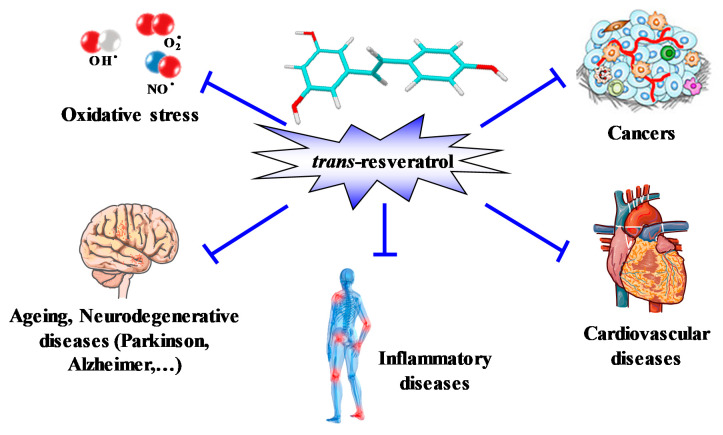
Pleiotropic action of RSV. The 3,4′,5-trihydroxystilbene is able to prevent coronary heart diseases, inflammatory pathologies, oxidative stress in aging, neurodegenerative diseases, as well as cancers.

Like many other plant polyphenols, RSV is considered to be a preventive food microcomponent in the same way as the flavonoids and epicatechins of green tea or cocoa [37]. In fact, RSV attracted little interest until 1992, when it was postulated to explain some cardioprotective effects of red wine, with interest then increasing from 1997 when Pezzuto’s team published a seminal paper reporting the ability of RSV to inhibit carcinogenesis at multiple stages [38]. Since then, the number of papers and citations have increased in an exponential manner due to the pleiotropic effects of RSV on various diseases (Figure 2). The main properties of RSV were found to relate to its antioxidant power, its anti-inflammatory properties, and its ability to modulate various targets in numerous pathologies (Figure 2) such as:

(1) Coronary heart diseases, via scavenging of reactive oxygen species (ROS) [39,40,41], prevention of the oxidation of low-density lipoproteins (LDL) [42,43,44], a decrease in foam cell formation and pro-inflammatory molecules [45,46], as well as a reduction in platelet aggregation [47,48,49].
(2) Cancers, through its ability to inhibit carcinogenesis at multiple stages (initiation, promotion, and tumor progression) in in vivo models of skin tumors [38]. Systemic administration of RSV has since been shown to inhibit the initiation and growth of tumors in a wide variety of rodent cancer models through various mechanisms, including cell cycle arrest, induction of apoptosis, and inhibition of angiogenesis (see for review [7,50]).
(3) Inflammatory diseases, where RSV is able to modulate both the adaptive and the innate immune response and consequently decrease the production of various pro-inflammatory cytokines in response to a wide range of exogenous stimuli [2,6].
(4) Age-related degenerative diseases, through its capacity to activate an important actor, sirtuin-1 (Sirt-1), which exhibits down-regulated expression in multiple organs during aging [51,52].

Therefore, these properties of polyphenol could participate in a chemopreventive or chemotherapeutic strategy against ocular diseases such as age-related macular degeneration (AMD), cataract, retinal degeneration, optic neuritis, glaucoma, and retinoblastoma through common mechanisms, as well as through specificities that characterize individual ocular diseases.

## 3. Age-Related Macular Degeneration and RSV Action

### 3.1. AMD and the Key Actors

AMD is one of the main causes of deterioration of vision in elderly people in developed countries [53], resulting in a loss of vision in the center of the visual field due to damage to the retina. It is usually classified as one of two forms: a dry form characterized by the appearance of drusens, which are proteinaceous collections at the level of the retinal pigment epithelium (RPE); and a wet form, in which neovascularization complicates retinal changes. At present, some therapies are used for wet AMD to inhibit the abnormal growth of blood vessels with VEGF inhibitors or laser photocoagulation, but a number of side effects to these therapies are seen, as is resistance. Concerning dry AMD, currently only nutritional supplementation is given; no therapies have shown efficacy. The aim of non-exudative AMD treatment is to delay the loss of visual function.

**Figure 3:**
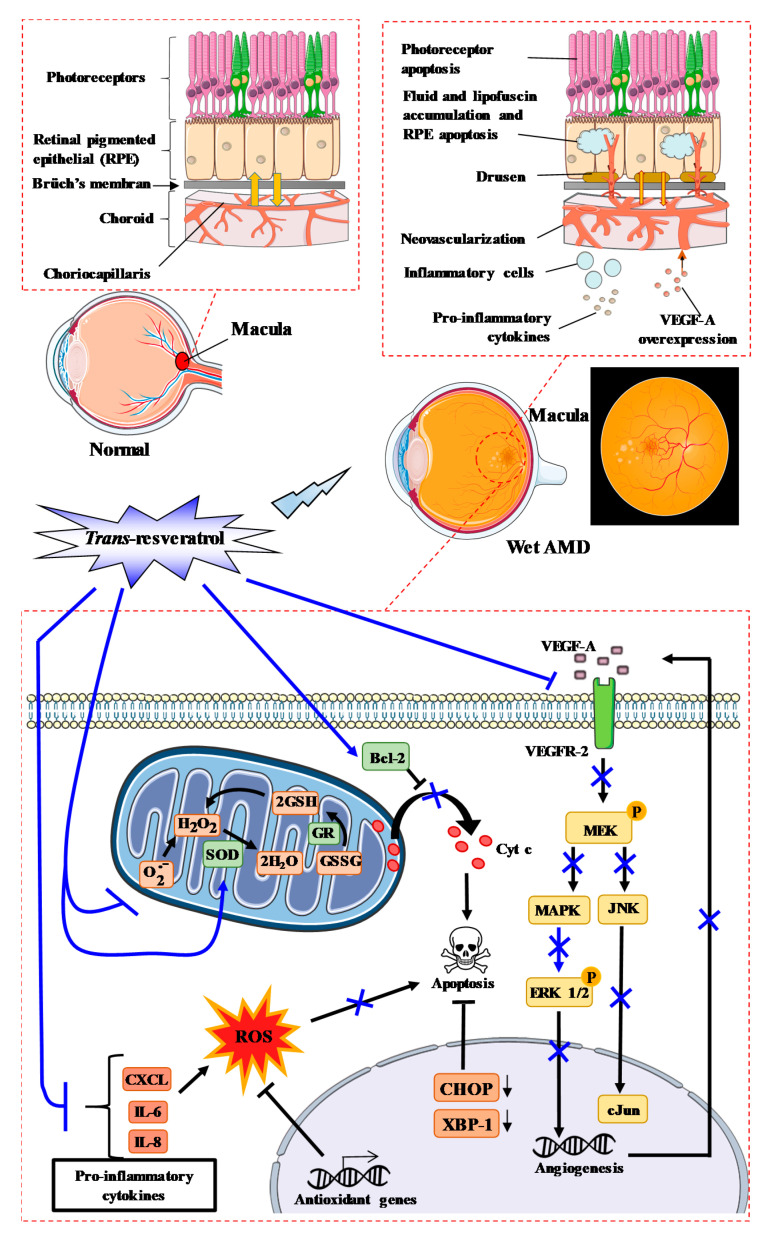
RSV could prevent oxidative stress involved in the development and progression of ocular diseases such as age-related macular degeneration (AMD). Various environmental factors produce free radicals, leading to oxidative stress in ocular tissues and consequently provoking the initiation of diseases such as AMD. On the one hand, RSV is able to scavenge free radicals (O_2_^−^) and activate superoxide dismutase (SOD) or glutathione reductase (GR), while on the other, it inhibits inflammation through the reduction of various pro-inflammatory cytokines and blocks the signaling pathway induced by vascular endothelial growth factor-A (VEGF-A) to promote neoangiogenesis.

Some therapies that modulate risk factors are able to prevent the development or progression of the pathology, but do not completely cure patients affected by AMD. Consequently, new therapies are needed and RSV could act on this disease at different levels. Indeed, keys processes have been identified in AMD such as oxidative damage, impaired activity or function of the RPE, increased apoptosis, and chronic inflammation (Figure 3). Moreover, neovascularization seems to play a very important role in AMD complications. RVS could act on these different steps mainly through its antioxidant power, anti-inflammatory action, or through its anti-angiogenic effects.

### 3.2. RSV and AMD Initiation

AMD is a multifactorial disease with both environmental and genetic risk factors [54]: air pollution, smoking, UV radiation, metabolic diseases (e.g., diabetes, hypertension, obesity), dietary fat consumption [55,56,57,58], and genetic polymorphisms such as *cfh*, *arms2/hrta1* genes [59]. Alone or in combination, these factors could contribute to initiating AMD through the production of free radicals such as superoxide anion (O_2_^·−^), nitric oxide (NO^·^), and hydroxyl radical (OH^·^), which create oxidative stress and inflammation in ocular tissues. It is now well-established that oxidative stress and inflammation play a critical role in the initiation and development of AMD [60].

Due to its antioxidant power and ability to scavenge free radicals, RSV could protect ocular tissues against oxidative stress (Figure 3). RPE cells, which form the cell layer responsible for maintaining retinal health by providing structural and nutritional support, are the primary target for AMD-associated oxidative stress [61]. Due to its antioxidant power, RSV could be effective at reducing the risk of AMD in the same manner as antioxidants commonly found in fruits and vegetables (vitamins C, E, and carotenoids) for which studies have demonstrated that their intake may delay or prevent the development of retinal diseases [62]. Indeed, the treatment of RPE cells with RSV prevents ROS production, both in the basal state (around 20% compared to untreated controls) and when cells are treated with an inducer of oxidative stress such as hydrogen peroxide (H_2_O_2_). Secondly, RSV protects or delays cell death induced by H_2_O_2_ in RPE cells. Indeed, King et al. have shown that the pretreatment of cells with RSV followed by treatment with H_2_O_2_ prevents the inhibition of cell proliferation [63]. Moreover, polyphenol protected against the oxidative damage of RPE cells by modulating SOD/MDA (malondialdehyde) activity and activating Bcl-2 expression [64]. These protective actions of RSV could involve the inhibition of mitogen-activated protein kinase (MAPK) pathways induced by oxidative stress. At the basal level, RSV was able to decrease the phosphorylation of extracellular signal-regulated kinase 1/2 (ERK 1/2), phospho-ERK1/2, in a dose-dependent manner, as well as the tyrosine/threonine mitogen-activated kinase kinase (MEK), especially with 25 and 50 µM RSV compared to cells treated with H_2_O_2_ alone [63]. This ability to reduce MAPK activation could contribute to a reduction in the effect of H_2_O_2_ on this pathway.

Other environmental factors, such as acrolein found in cigarette smoke, induce oxidative stress in human retinal cells. A study performed at the ophthalmology department in Taiwan showed that RSV pretreatment can also prevent damage caused by exposure to acrolein for 7 days followed by H_2_O_2_ treatment [65,66]. In the same way, other studies have shown that RSV, alone or in combination with polyunsaturated fatty acids (PUFA; Resvega^®^), was able to improve RPE cell viability upon cigarette smoke-derived hydroquinone exposure and reduce the release of interleukin (IL)-8 and monocytic chemoattractant protein (MCP)-1 from RPE cells [67,68]. Under these conditions, RSV upregulated C/EBP homologous protein (Chop) and spliced X-box binding protein 1 (XBP1), thereby improving mitochondrial bioenergetics, upregulating antioxidant genes, and stimulating the unfolded protein response [68,69].

UV radiation, especially UVA, can also penetrate the lens, reach the retina, and induce oxidative stress in RPE cells. RSV was able to: (i) reduce the UVA-induced decrease in RPE cell viability, (ii) reduce the generation of intracellular H_2_O_2_, and (iii) decrease the activation of UVA-induced extracellular signal-regulated kinase, c-jun-NH_2_ terminal kinase, and p38 kinase in RPE cells, as well as cyclooxygenase-2 (COX-2) expression [70].

In a similar manner at our laboratory, we have shown that the toxic effects of oxysterols, which come from the diet or from cholesterol catabolism and play important roles in AMD, could be counteracted by RSV [71]. Indeed, when retinal cells (ARPE-19) were treated with 7-beta-hydroxycholesterol (7β-OH) or with 7-ketocholesterol (7KC), we observed a decrease in viable cells after 24 h and more strongly after 40 h. RSV, which has no toxic effect on retinal cells, can protect these cells from the toxic effects of oxysterols.

Other antioxidant mechanisms of RSV may be involved. Indeed, we have shown in various cell lines that RSV could act on ROS production via its action on mitochondrial enzymatic pathways [7].

RSV can interfere with mitochondrial electron transport and promotes a fall in the mitochondrial transmembrane potential Δψ_m_ [72,73], a decrease in ATP production, and the generation of ROS (Figure 3) [74]. RSV has also been described to: (1) decrease complex III activity by competing with coenzyme Q, whose complex is the site of ROS production, (2) inhibit ATPase activity, and (3) scavenge superoxide anions [75]. Furthermore, O_2_^−^ radicals are converted to H_2_O_2_ by superoxide dismutase (SOD), and H_2_O_2_ detoxification normally occurs according to two different reactions, i.e., either by the thioredoxine reductase (TR) or by reacting with glutathione reductase (GSH) and glutathione peroxidase (GPx). The latter reaction produces water and oxidized glutathione (GSSG), and GSSG is recycled to GSH by glutathione reductase (GR). Therefore, in addition to scavenger O_2_^−^, RSV accelerates the detoxification of O_2_^−^ by inducing an increase in glutathione levels and also by inducing GPx, GR, glutathione-S-transferase (GST), and catalase (CAT) activities [76,77,78,79,80], as well as their mRNAs levels [81]. This modulation of antioxidant enzymes could explain the inhibition of DNA damage in human lymphocytes induced by various toxic drugs (i.e., H_2_O_2_, 1,2-dimethylhydrazine, bleomycin) [82,83,84].

Metabolic diseases can also play an essential role in the production of oxidative stress. It is now well-defined that diabetes and obesity have numerous effects on ocular diseases [85]. A wide range of evidence implies that dietary hyperglycemia is etiologically related to human aging and diseases, including DR and AMD. In this context, these diseases can be considered as retinal metabolic diseases. A number of clinical trials have been published investigating the effects of RSV on whole-body energy metabolism in relation to the multiple health factors that are affected by obesity and type 2 diabetes [86,87,88,89,90]. Indeed, daily administration of 2.5 or 5 g RSV for 28 days shows a decrease of fasting and postprandial glucose and insulin. At low concentrations such as 5 mg twice daily for 4 weeks, RSV significantly decreased insulin resistance [91]. Timmers et al. showed that 75 mg of RSV twice daily for 30 days improved the metabolic profile in a healthy obese man: RSV reduced sleeping and resting metabolic rates [88]. In muscle, RSV activated the AMPK–SIRT1–PGC1α axis, reduced blood glucose and insulin levels, reduced liver fat, improved muscle mitochondrial function, and reduced inflammatory markers in the blood [88]. Consequently, through the pleiotropic action of RSV on metabolic diseases, this polyphenol could contribute to reducing the collateral effects of metabolic diseases on eye disorders, especially AMD.

A final and important mechanism in this step of AMD initiation is the accumulation of lipofuscin or cellular debris in RPE. Lipofuscin, an aging pigment, is considered to be a credible marker for the aging of cells. Lipofuscin tends to accumulate even at an early age, but rapidly progresses with the advancement of the aging process, suggesting the inability of autophagy to handle the waste disposal capacity. The decline of autophagy during aging appears to be the cause for lipofuscin accumulation. Morselli *et al.* showed that transgenic expression of Sirt-1 induces autophagy in human cells *in vitro* and in *C. elegans* in vivo [92]. Caloric restriction and RSV promotes longevity through the Sirt-1-dependent induction of autophagy [93]. Moreover, RSV induced autophagy in ARPE-19 cells, as determined by the increased presence of autophagic vacuoles, increased the LC3II/I ratio, and decreased p62 expression [94]. In this study, RSV acted similarly to proteasomal inhibition downstream of the mammalian target of rapamycin (mTOR), since upstream inhibition of autophagy by 3-methyladenine was not able to inhibit autophagy in ARPE-19 cells. This effect on autophagy is also found with a combination between RSV and PUFA (especially omega-3), where 288 ng of Resvega^®^, containing 30 mg of *trans*-RSV and 665 mg of omega-3 fatty acids, among other nutrients, was able to induce autophagy and contribute to the survival of ARPE-19 cells exposed to detrimental protein waste triggered by proteasome inhibition [95].

RSV was also able to prevent the toxic effects of N-retinyl-N-retinylidene ethanolamine (A2E), a major lipofuscin component that accumulates in RPE cells with age. Indeed, polyphenol pretreatment strengthened cell monolayer integrity through the preservation of trans-epithelial electrical resistance, maintained the intracellular redox balance, and prevented A2E-induced mitochondrial network fragmentation [96]. Moreover, RSV and its metabolite, piceatannol, reduced intracellular A2E accumulation in RPE cells [97].

### 3.3. RSV and Inflammatory Processes Related to AMD

We and others have shown that RSV is an efficient anti-inflammatory compound in various models of cardiovascular disease, cancer, and chronic inflammatory diseases [5,50,98]. Inflammation also plays a central role in ocular pathologies, especially in AMD [99,100,101], and thus the use of RSV to counteract inflammatory processes could be relevant herein.

Based on the important role played by interleukins, a study has shown that RSV was able to reduce the production of interleukin-6 (IL-6) and interleukin-8 (IL-8) induced by glucose in retinal cells [102]. This latter interleukin, IL-8, has been shown to be an important risk factor for AMD [101]. Furthermore, RSV substantially inhibited pro-inflammatory cytokine-induced CXCL11 production, which is an important chemokine involved in inflammatory cell recruitment [103]. Indeed, RPE cells adjacent to drusen deposits in the AMD eye are known to contain CXCL11. This is not the only chemokine modulated by RSV—this polyphenol was also able to inhibit the cytokine-induced expression of chemokines CXCL9, CCL2, and CCL5.

Another mechanism could involve peroxisome proliferator-activator receptors (PPAR). Indeed, RSV protects RPE cells from sodium iodate injury (increasing levels of ROS and IL-8) through the activation of PPARα and alteration of PPARδ conformation [104].

### 3.4. RSV Can Prevent Complications of AMD

The ultimate step in the AMD process is neovascularization, which constitutes a major complication of AMD. The molecular mechanisms involve vascular endothelial growth factor A (VEGF-A) and an increase in vascular permeability that results in loss of vision [105]. Anti-angiogenic therapies targeting VEGF have proven to be highly effective in treating neovascular AMD, but they cause a number of side effects. In this approach of angiogenesis inhibition, RSV could counteract AMD through its action on VEGF contributing to the abnormal growth of blood vessels. To test this hypothesis, we tested the protective effect of RSV on the VEGF secretion induced by oxysterols in RPE cells [71]. Oxysterols induced VEGF-A secretion after 24 and 40 h of treatment with 7β-hydroxycholesterol and 25-hydroxycholesterol. Interestingly, cotreatment with RSV at 1 µM decreased VEGF-A secretion induced by these oxysterols both at 24 and 40 h [71].

Consequently, RSV could reduce neovascularization or protect against various factors that induce VEGF-A production and promote neovascularization such as diabetes, which is known to induce VEGF and which is linked to the progression of vision loss (see below). In this regard, Kim et al. have shown, in retinal tissues, that RSV pretreatment can prevent diabetes-induced rises in VEGF secretion [106]. Notably, they observed an increase in VEGF levels between the outer plexiform layer and the nerve fiber layer on retinal sections of mice 2 months after the induction of diabetes compared to control mice. Indeed, the authors showed that RSV pretreatment of mice by oral gavage at 20 mg/kg once a day for 4 weeks prevents VEGF secretion as well as VEGF expression. Moreover, RSV was able to reduce both epidermal growth factor-A (EGF-A) and VEGF-C secretion by human RPE cells stimulated with a mixture of inflammatory cytokines (interferon [IFN]-γ, tumor necrosis factor [TNF]-α, IL-1β) and to decrease tumor growth factor (TGF-β) and cobalt chloride [107]. This potential effect on hypoxia has been raised in several other studies where RSV inhibited hypoxic choroidal vascular endothelial cell proliferation through activation of the stress-activated protein kinase (SAPK)/Jun amino-terminal kinase (JNK) pathway [108]. The development of choroidal neovascularization (CNV) is a critical step in the pathogenesis of AMD, and laser photocoagulation has been described to induce CNV in mice. A preclinical study has shown that CNV volume was significantly lower in the RSV-treated mice 1 week after laser treatment compared with vehicle-treated animals. Very interestingly, RSV inhibited macrophage infiltration into RPE–choroid and suppressed the expression of inflammatory and angiogenic molecules, including VEGF, monocyte chemotactic protein-1 (MCP-1), and intercellular adhesion molecule-1 (ICAM-1) [109]. The underlining molecular mechanism appeared to involve the maintenance of adenosine monophosphate-activated protein kinase (AMPK) levels, this latter exerting its inhibitory effect on the nuclear factor-κB (NFκB) in the RPE–choroid complex.

## 4. Glaucoma and RSV Action

### 4.1. Glaucoma and the Key Actors

Glaucoma is a progressive optic neuropathy defined by damage to the optic nerve associated with the degeneration of retinal ganglion cells (RGC), causing visual field damage and subsequent blindness. Ocular hypertonia (OH) is the first known risk factor that contributes in most cases to the pathology and remains to date the only pathogenic event accessible to medical or surgical treatment. Glaucoma is the second leading cause of blindness in the world after cataracts. It has been projected to affect 80 million people worldwide by 2020 [110] and 112 million people by 2040 [111]. The pathological elevation of intra-ocular pressure (IOP) is due to alteration and then degeneration of the trabecular meshwork. Aqueous humor (AH) is eliminated at a rate of 90% by the trabecular route through the trabeculum and at a rate of 10% by the so-called uveoscleral route. The trabeculum is considered a dynamic filter that drains AH out of the anterior chamber of the eye. It is located in the iridocorneal angle over its entire circumference. Therapy is based on either medical, laser, or surgical intervention. However, recent works tend to show that a nutritional prevention strategy could be interesting. Indeed, certain lifestyle habits could influence glaucoma progression or its prevention. For exemple, frequency of coffee intake may be associated with disease progression [112].

### 4.2. RSV and the Oxidative Stress in Glaucoma

**Figure 4:**
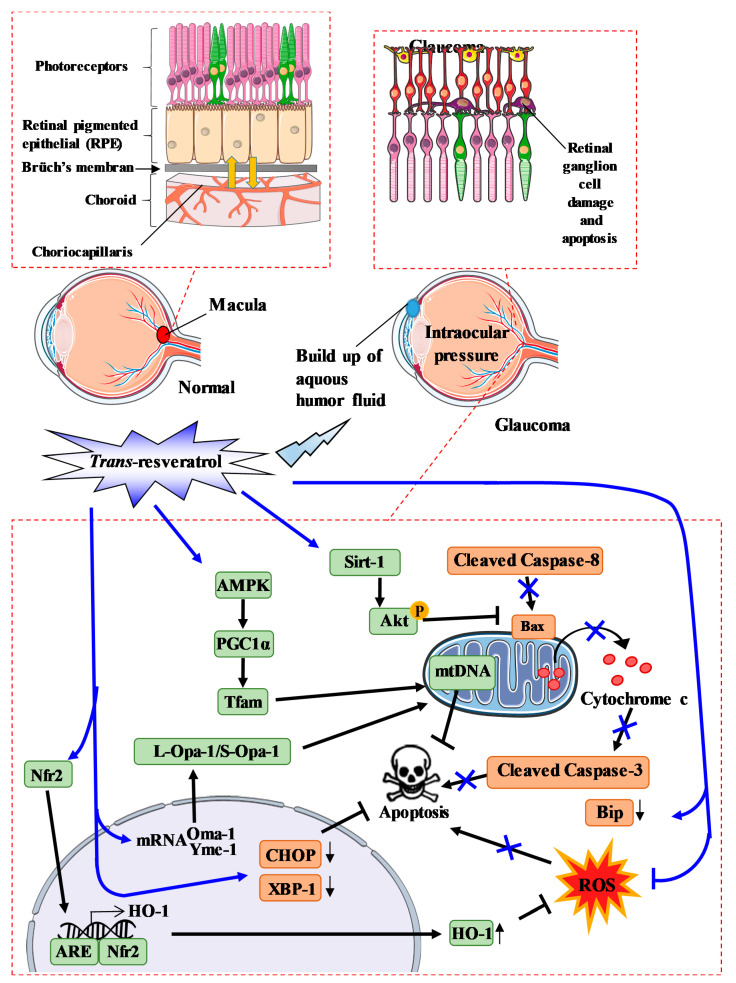
Proposed model of resveratrol action during oxidative stress in glaucoma. *Trans*-resveratrol (RSV) activates sirtuin-1 (Sirt-1), which leads to the phosphorylation of Akt and inhibits Bax activity. RSV also activates the AMPK pathway, which leads to mitochondrial DNA transcription and replication. RSV is also able to alter Oma-1 and Yeme-1 mRNA expression, leading to the alteration of the L-Opa-1/S-Opa-1 (long-optical autophagy 1/short optical autophagy) ratio. In addition, RSV also lowers ROS levels not only by activating the scavenger pathway, but also by facilitating the translocation of Nrf2 into the nucleus, thereby favoring interaction between Nfr2 and the antioxidant response element (ARE) leading to HO- production.

Oxidative stress is known to be an early event in hydrostatic pressure-induced RGC damage involved in glaucoma. Indeed, several studies have revealed that oxygen metabolism, and more particularly, ROS are crucial in the development of glaucoma. As described previously, RSV has antioxidant properties that, through its hydroxyl groups, have the ability to react with ROS in glaucoma [113]. Nevertheless, this is not RSV’s only antioxidant ability. In fact, many studies have demonstrated that RSV can reduce ROS in glaucoma models (in vitro and in vivo) through several pathways (Figure 4) [114,115,116]. This downregulation of ROS could be due to the modulation of the endogenous antioxidant system [117], such as induction of nuclear factor erythroid-2-related factor 2 (Nrf2) translocation into the nucleus, where it binds to antioxidant response elements (ARE). This latter leads to the production of heme oxygenase-1 (HO-1), an anti-oxidant enzyme [117,118].

Another important pathway regulating ROS production is the mitochondrial pathway. Indeed, mitochondria are crucial organelles in glaucoma, and oxidative stress can be produced by an imbalance between ROS production and ROS defenses. A significant decrease in the activity of detoxifying enzymes such as SOD and GPx has been shown in the aqueous humor of patients with glaucoma [119]. Thus, targeting mitochondrial homeostasis could be a potential therapy for glaucoma. Some studies have shown that RSV can alter not only the quantity but also the quality of mitochondria in RGC. Indeed, it has been shown that RSV is able to increase the number of mitochondria present in one cell [120] and can affect pathways leading to mitochondrial biogenesis. In this way, RSV is able to downregulate the Oma-1 gene and upregulate the Yeme-1 gene, which increase the ratio L-Opa-1/S-Opa-1 (long-optical autophagy 1/short optical autophagy 1) involved in mitochondrial fusion (Figure 4) [121]. Moreover, RSV is able to restore protein levels that are decreased in glaucoma and which are involved in mitochondrial biogenesis, such as AMPK, peroxisome proliferator-activated receptor γ coactivator-1α (PGC-1α), and mitochondrial transcription factor A (Tfam), which leads to mitochondrial DNA transcription and replication [115]. Zhang et al. have also shown that RSV modifies not only the number but also the quality of mitochondria by maintaining mitochondrial integrity. Indeed, RSV are able to decrease the membrane potential depolarization induced by pressure.

### 4.3. RSV and the Cell Death Process in Glaucoma

RGCs are the main cell type affected in optic neuropathies, with degeneration of these cells leading to profound phenotypes and eventual loss of vision. It has been reported that the pathways of RGC loss have been explored in depth after acute high IOP injury, such as apoptosis, autophagy, and necrosis. Furthermore, pyroptosis, a novel type of pro-inflammatory cell programmed necrosis, plays a crucial role in retinal neuronal death, especially in the ganglion cell layer, by acute high IOP injury that peaks at 6 h [122]. Several other in vitro and in vivo studies have shown that RSV treatment or pre-treatment before inducing stress reduces cell apoptosis. For instance, in various glaucoma models, RSV has been demonstrated to reduce the activation of caspase-3, as evidenced by the decrease in cleaved caspase-3 protein expression levels [121,123,124]. In a mouse model of ischemia/reperfusion (I/R) injury, RSV was shown to alter the extrinsic apoptosis pathway by reducing caspase-8 mRNA levels and proteolytic cleavage in mice retina [123]. Furthermore, other studies have investigated the mitochondrial apoptosis pathway, and showed that RSV can also reduce the expression of Bax and decrease cytochrome c release. Similarly, another study has been carried out to investigate the effect of RSV on the endoplasmic reticulum (ER) stress apoptosis pathway [125]. This study revealed that RSV reduces the level of cytosolic Bip and also reduces the nuclear quantity of XBP-1 and CHOP, which consequently reduces apoptotic rates [125]. In a preclinical study, C57BL/6J male mice were injected with RSV for 2 consecutive days before I/R retinal injury, which induces apoptosis in RGCs. In this model, RSV treatment significantly reduced the loss of retinal morphology and downregulated mRNA expression and activation of caspase-8 and caspase-3 protein and subsequently reduced apoptosis [123]. These observations were also confirmed in vitro by others [126].

### 4.4. RSV and the Inflammatory Process in Glaucoma

Neuro-inflammation in glaucomatous pathology is a component due to the role of immune and glial cells in the early stages of the pathology. In fact, astrocytes, microglia, and monocytic cells are defined as being critical players in the neuro-inflammatory response in glaucoma. Therefore, a number of studies have investigated the potential inflammatory effect of RSV on trabecular meshwork (TM) endothelial cells. Indeed, it has been shown that the induction of the inflammatory markers IL-1α, IL-6, IL-8, and endothelial leucocyte adhesion molecule-1 (ELAM-1) in TM cells subjected to chronic oxidative stress is dependent on the activation of intracellular ROS (iROS) generated by the mitochondria. RSV could be used to reduce the iROS production that results from oxidative stress. A study recently demonstrated that RSV could be used to lower IL-1α levels. In this study, authors showed that IL-1α levels were unrelated to those of ELAM-1 [127]. This suggests that other mechanisms could be affected in glaucomatous TM cells. Furthermore, Luna et al. showed that RSV prevents inflammatory markers such as IL-6 and IL-8 in TM cells under chronic oxidative stress conditions [128]. Another mechanism that could be involved is the activation of adenosine A1 receptors, which may lead to increased matrix metalloproteinase 2 (MMP-2) activity. Razzali et al. have shown that the repeated topical application of RSV for 21 days in steroid-induced ocular hypertensive (SIOH) rats significantly reduces IOP and TM thickness [129]. In these in vivo experiments, RSV also significantly increased ganglion cell layer thickness, the linear cell density in these cells, and inner retinal thickness. Furthermore, it also significantly reduced retinal oxidative stress compared to the SIOH vehicle-treated group. Interestingly, pretreatment with an A₁ antagonist abolished the oculohypotensive effect of RSV, suggesting that its oculohypotensive action involves its agonistic activity at the A1 adenosine receptor [130]. In a model of IOP-induced retinal ischemia in rats, RSV was able to reduce detrimental effects due to ischemia through a potential downregulation of MMP-9 and inducible nitric oxide synthase (iNOS), as well as through the upregulation of HO-1 [116].

## 5. Cataract and RSV Action

Cataract is the partial or total clouding of the lens. It is a ubiquitous condition that affects an increasing number of people around the world every year. The risk factors of cataract development are similar to those of AMD and include a wide range of lifestyle parameters (i.e., smoking, alcohol, fatty diet, stress, etc.), although there are also rarer etiologies, as well as traumatic and congenital factors. Furthermore, the main risk factors for age-related cataracts are glaucoma and diabetes, which can lead to secondary cataracts.

The extent of cataract formation significantly increases with age in *ad libitum-*fed mice. Strikingly, this increase was attenuated by RSV treatment, which was more effective than in the every-other-day-feeding group at 30 months of age [131]. In an experimental model of naphthalene (1 g/kg/day, po)-induced age-related cataract in rats, RSV (20 and 40 mg/kg/day, i.p.) retarded lenticular opacity, restored antioxidants (CAT, SOD, GPX, GSH), Ca^2+^ ATPase function, protein contents, and reduced lipid peroxidation in the lenses of RSV-treated rats [132]. RSV was able to significantly inhibit the TGFβ2-induced expression of the myofibroblast marker, α-SMA, in a human lens cell line (FHL124) and human capsular bags following simulated cataract surgery, indicating the ability of RSV to prevent the EMT associated with posterior capsule opacification (PCO). [133]. Interestingly, in cultured lens epithelial cells, RSV inhibited apoptosis and decreased acetyl-p53 levels under oxidative stress induced by H_2_O_2_ [134].

## 6. Diabetic Retinopathy and RSV Action

DR is one of the most common complications of type I and type II diabetes. About 80% of patients who have lived with diabetes for 15 years have clinical signs of retinopathy and more than 10% of them are affected by vision loss. This latter is the consequence of slow and progressive alterations in the microvessels of the retina, leading to the opening of the blood–retinal barrier (BRB), the pathological proliferation of blood vessels, and the formation of fibrous tissue in the vitreous cavity, resulting in retinal detachment. During this pathology, neuroglial dysfunction, significant oxidative stress, as well as inflammation and angiogenesis are found. In addition, an increase in the permeability of the retinal vasculature is observed, as well as migration of leukocytes into the retina. Moreover, alterations in biochemical pathways, such as increased flux of advanced glycation end products/receptors (AGE/RAGE), the polyol pathway, protein kinase C (PKC) activation, and the hexosamine pathway induced by hyperglycemia, produce oxidative stress and cause the rupture of the BRB, pericyte loss, and increased vascular permeability [135]. At present, the recommended treatment for severe non-proliferative and proliferative DR is photocoagulation and intravitreal injections of anti-VEGF, with or without focal laser treatment for diabetic macular edema.

**Figure 5:**
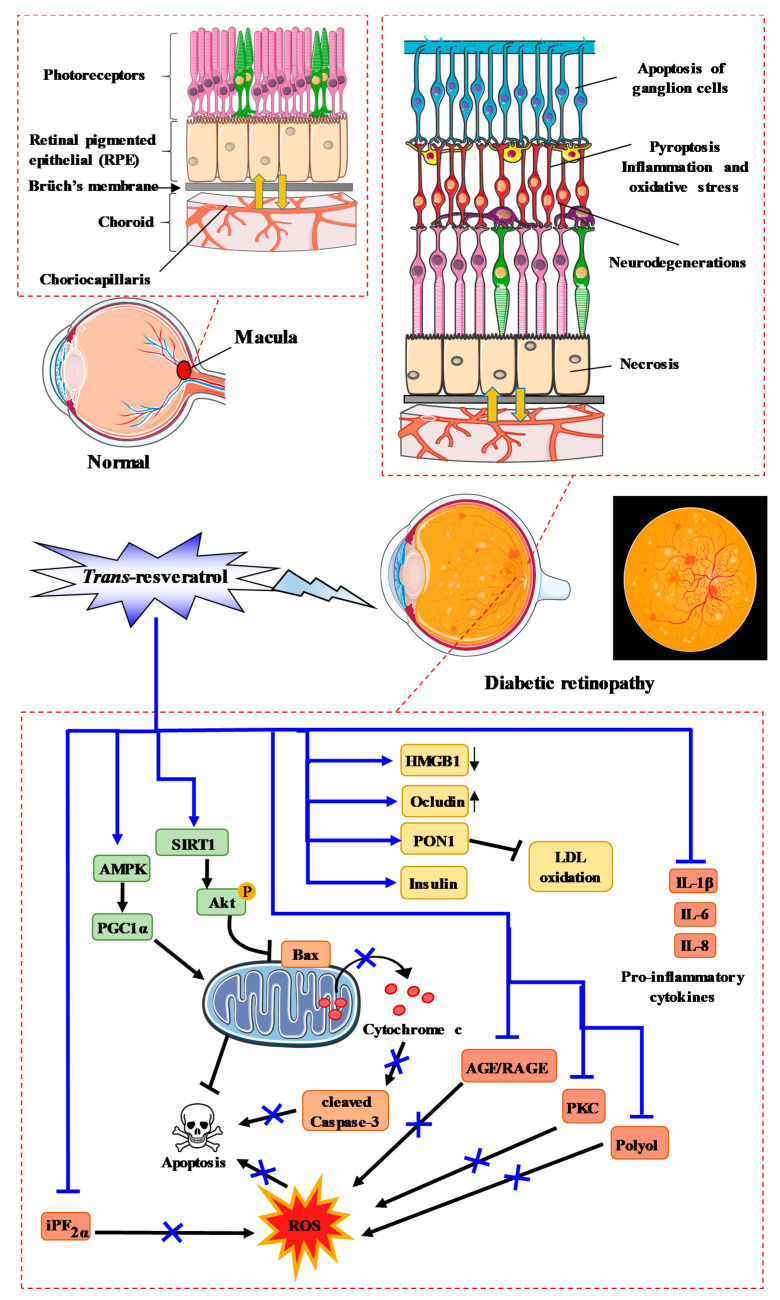
RSV action and process of diabetic retinopathy. RSV activates sirtuin-1 (Sirt-1), which leads to the phosphorylation of Akt and inhibits the mitochondrial apoptosis pathway. Furthermore, RSV stimulates mitochondrial activity through the AMPK–PGC1α pathway, which reduces apoptosis. RSV also lowers ROS through the iPf2α, AGE/RAGE, PKC, and polyol pathways, and downregulates pro-inflammatory cytokines such as IL-1β, IL-6, and IL-8.

RSV is able to prevent oxidative stress due to its pleiotropic actions on various key actors. Indeed, RSV was shown to be able to significantly decrease ROS production by increasing retinal SOD activity, which is one of the most important antioxidant defense systems in the retina [136,137,138]. Conversely, RSV decreased retinal 8-*iso*-prostaglandin F_2*α*_ (iPF_2*α*_), a marker of oxidative damage, and reduced glutathione levels [138,139,140]. Very interestingly, RSV was able to affect the MAP kinase pathway by inducing phosphorylation of protein kinase B (Akt), for which a reduction is observed in retinal neurodegeneration [138,141], phosphorylation of AMPK, Sirt-1 expression, and PGC-1α protein expression [137], and decreasing phosphorylation of ERK 1/2 (Figure 5) [138]. Consequently, RSV increased the thickness of both the whole retina and the inner nuclear layer in diabetic rats [138].

RSV significantly attenuated diabetes-induced downregulation of occludin and diabetes-induced upregulation of high-mobility group box-1 (HMGB1), as well as the receptor for advanced glycation end products and BRB breakdown in diabetic retina [142].

Very interestingly, RSV restored the insulin level and paraoxonase 1 (PON1) expression and activity, as well as clearly reducing retinal vascular permeability, retinal AGEs, low-density lipoprotein (LDL), oxidized LDL (Ox-LDL), caspase-3 activity, and retinal damage in STZ-induced diabetic rats [139,143]. Moreover, in the same model, RSV decreased various pro-inflammatory cytokines such as IL-1β, IL-6, TNFα, VEGF, IFNγ, MCP-1 [143], and NFκB, as well as TNFα and apoptosis [144]. In this pathway involving NFκB, RSV prevented the increase in p65 acetylation, the binding of p65 at the metalloproteinase MMP-9 promoter, and MMP-9 activation, as well as mitochondrial damage and retinal endothelial cell apoptosis [145]. In another model using high-glucose (HG)-stimulated rat retinal endothelial cells (RRECs), RSV reduced caspase-3 activity and Ox-LDL expression [143]. Thus, hyperglycemia and oxidative-osmotic nitrosative stress play a role in the pathophysiology of diabetic cataract (Figure 5). RSV in combination with nicotinamide exhibits strong effects; it was shown that RSV (10 or 30 mg/kg/day) was not able to prevent the onset of diabetic cataract, but significantly delayed its progression compared to the control group [146].

Mechanistically, the delayed progression due to RSV was shown to be linked to a decrease in protein carbonyl levels in diabetic lenses [146], to the inhibition of the aldose reductase enzyme, and to the reduction in the formation of glycation end products in rat lens [147]. Moreover, in a glucose-induced lens opacity model, RSV displayed a protective effect by preventing opacification in cattle lens. These results suggest that RSV partially delays diabetic cataracts through the attenuation of oxidative damage to lens proteins [146]. This prevention of oxidative damage in lens epithelial cells seemed to be mediated by various processes, such as SOD-1, HO-1, CAT, forkhead box O (FoxO) activity, in particular FoxO1A, FoxO3A, and FoxO4, or inhibition of P38 and JNK phosphorylation [148,149].

Very interestingly, RSV at a dose of 40 mg/kg/day (i.p. injection) in a model of naphthalene (1 g/kg/day, po)-induced age-related cataract (ARC), prevented cataract formation associated with aging through an increase in soluble proteins and Ca^2+^ homeostasis (i.e., Ca^2+^ ATPase pump activities in the lens) [132].

By acting on the key actors of the cellular matrix, RSV could prevent posterior capsule opacification (PCO), which is a common complication of cataract surgery. Indeed, RSV was able to inhibit suppressed expression of TGF-β2-induced genes associated with fibrotic disease, including in the treatment of STZ-induced diabetic cataract [150]. In general, the cellular pathways induced by DR lead to apoptosis of retinal cells. RSV was able to suppress STZ-induced apoptosis of retinal cells in the inner nuclear layer of retina after administration of 10 mg/kg/day for 7 months.

Very interestingly, these beneficial effects were inhibited by the miR-29b inhibitor [151]. RSV inhibited the high-glucose-induced decreases in glutamate uptake, glutamine synthase activity, glutamate transporters, and glutamine synthase expression in high-glucose-treated Müller cell cultures compared to controls [152]. Moreover, RSV (10 mg/g/day) alleviated hyperglycemia in diabetic rats and attenuated diabetes-induced decreases in the amplitude of a-wave in rod response, decreases in the amplitude of a- and b-waves in the cone and rod response, and decreases in amplitude of OP2 in oscillatory potentials [152].

Immune cells seem to be involved in the inflammatory process found in DR through the release of IL-17 by lymphocyte T helper 17 (Th17). Indeed, serum of patients with DR exhibit higher IL-17 expression than non-diabetic control groups [153,154]. Moreover, peripheral blood mononuclear cells (PBMCs) exhibited increased expression levels of IL-17 in the DR group of patients, whereas Sirt-1 protein expression decreased in the PBMCs of patients with DR [154]. Interestingly, RSV was able to reduce IL-17 secretion levels in PBMC cultures from patients with proliferative DR, as well as restore Sirt-1 expression in both genic and protein expression [154]. These results obtained on cultured PBMCs echo the results that we have been able to demonstrate directly on Th17 immune cells, where resveratrol was able to reduce the production of IL-17, as well as alter the process of differentiation of naive T lymphocytes into pro-inflammatory Th17 immune cells [155]. In addition, in numerous in vivo models, we have been able to show that resveratrol is able to decrease not only the secretion of IL-17, but also the key factors involved in lymphocyte differentiation. This process is associated with a highly significant decrease in angiogenesis by reducing VEGF-A secretion. This mechanism is dependent on the Sirt-1 protein, where the conditional invalidation of Sirt-1 in T CD4 cells demonstrated that this effect on the production of IL-17 and VEGF was under the control of the Sirt-1 protein and the nuclear transcriptional factor STAT3 [155].

## 7. Vitreoretinopathy and RSV Action

Proliferative vitreoretinopathy (PVR) is the main cause of failure following retinal detachment surgery. The membrane cells seen in PVR are of the pseudofibroblastic type, but they have several origins, in particular astrocytes and RPE cells. The importance of RPE cells in VRP likely stems from the access of RPE cells to the vitreous due to retinal invasion and the dispersion of viable RPE cells into the vitreous during cryopexy treatment of retinal tears. There are various key actors involved in the development of PVR, which is dependent on the migration and proliferation of RPE cells, glial cells, and inflammatory cells [156]. In this way, TGF-β2-induced epithelial-to-mesenchymal transition (EMT), and EMT inhibition decreases collagen gel contraction and fibrotic membrane formation, resulting in the prevention of PVR. Moreover, numerous cytokines such as IL-1β and IL-6 are considered to be key factors in the development of PVR.

In a model of ARPE-19 cells, RSV suppressed the decrease of zona occludens-1 (ZO-1) and caused an increase of alpha-smooth muscle actin expression in TGF-β2-treated cells and increased vimentin expression [157]. Very interestingly, RSV decreased TGF-β2-induced wound closure and cell migration, as well as collagen gel contraction and the phosphorylation of Smad2 and Smad3 in TGF-β2-treated ARPE-19 cells [157]. With regard to Smad protein, another study in RPE cells has shown that RSV induced EMT and inhibited TGF-β2-induced EMT of RPE cells by deacetylation of Smad4 [158]. TGF-β2 is not the only growth factor involved: platelet-derived growth factor (PDGF) has been shown to enhance the proliferation and migration of RPE cells in PVR. RSV was also able to inhibit PDGF-BB, an isoform of PDGF and PI3K/Akt, ERK, and p38 pathways, but had no effect on RPE cell adhesion to fibronectin [63,159].

## 8. Corneal Infection and RSV Action

Corneal infection represents a group of serious and blind ocular diseases. The cornea, in its normal condition, is highly resistant to microbial invasion. Nevertheless, pathogens may invade the cornea if epithelial integrity is breached. Corneal infection is a common ocular infection that can lead to ocular morbidity and blindness. These infections can be caused by several bacteria, virus, or fungi, with the common treatment being antibiotic drops.

Some studies have investigated the potential antibiotic action of RSV on corneal infection. An in vivo study revealed that RSV can reduce the cytotoxicity of *Acanthamoeba castellanii* on the HBMEC cell line [160]. Marino et al. showed that RSV can have a beneficial effect on *Staphylococcus aureus*-induced keratitis in an ex vivo culture model of rabbit cornea [161]. Indeed, this study demonstrated that RSV treatment can downregulate cell surface TLR2 on cells and reduce the expression of the interleukin-8 gene and, therefore, reduce the action of *Staphylococcus aureus*. In order to better understand the action of RSV on corneal infection, Tsai et al. investigated its antioxidant action on a human corneal epithelial cell line exposed to levofloxacin or moxifloxacin. They demonstrated that RSV not only increased cell survival, but also reduced intracellular accumulation of oxidative stress [162]. Unfortunately, the mechanism of RSV on corneal infection remains poorly understood, and further studies are required in order to gain deeper insight into the mechanism of RSV on corneal infection.

## 9. Potential Epigenetic Action of RSV in AMD and Cataract Prevention

RSV could also act by other mechanisms, particularly through sirtuins, which have recently been described as essential targets in various processes such as cellular stress resistance, genomic stability, tumorigenesis, and energy metabolism [8]. Sirt-1 is found in various organs and tissues regulating a variety of pathways such as glucose production in liver, fat mobilization and lipid metabolism in adipose tissue, as well as angiogenesis in blood vessels or other processes in the brain, pancreas, and intestine. Sirt-1 is expressed in almost all ocular tissues, including the cornea, lens (epithelial cells), iris, ciliary body, and retina. Particularly in the cornea, Sirt-1 is located in the corneal epithelial cells, keratocytes, and corneal endothelial cells. In the retina, Sirt-1 is found in the RPE, outer nuclear layer (ONL), inner nuclear layer (INL), and ganglion cell layer (GCL).

In eyes, Sirt-1 seems to protect the retinal cells from DNA damage such as oxidative stress-related retinal damage, apoptotic retinal death, and anti-inflammation. In addition, the breakdown of Sirt-1 causes retinal damage through multiple mechanisms. Thus, RSV, which is described as an agonist of sirtuins, could protect the eye by Sirt-1 activation. In this way, Kubota et al. showed that oral RSV pretreatment at a dose of 50 mg/kg for 5 days reverses retinal Sirt-1 activity to restore its basal activity in BALB/c mice exposed to 5000-lux white light for 3 h [163]. These events are associated with a significant decrease in the number of apoptotic cells in the ONL. Moreover, recent studies have shown that RSV inhibits hypoxia-inducible factor (HIF)-1α accumulation and VEGF secretion induced by cobalt chloride (CoCl2) through Sirt-1 in human RPE cells [108,164]. In this way, RSV downregulated VEGF-R2 phosphorylation and activation induced by VEGF in endothelial cells via Sirt-1 and, thus, could contribute to reducing choroidal neovascularization. Furthermore, RSV restored the activity of DNA methyltransferases (DNMTs), which catalyze the methylation process, in ARPE-19 cells, and, more specifically, restored LINE-1 methylation in retinal cells under oxidative and inflammatory conditions [165]. These results are not surprising, partly since Sirt-1 regulates the activity of DNMTs, especially DNMT1 [166]. The overexpression of Sirt-1 is associated with an induction by RSV of AMPK phosphorylation and an overexpression of PGC-1α protein [137]. Indeed, the beneficial effects of RSV were suppressed by inhibitors of AMPK and Sirt-1, as well as by treatment with PGC-1*α* siRNA. This AMPK pathway is also demonstrated in a mice model of STZ-induced diabetes [167]. Nevertheless, Michan et al. showed that neither overexpression of Sirt-1 by a genetic approach (i.e., conditional Sirt-1 overexpression in retinal neurons or vessels by breeding Sirt-1 overexpressing flox mice with Nestin-Cre or Tie2-Cre mice, respectively) or by a pharmacological approach with Sirt-1 agonists (i.e., RSV) provides additional protection against retinopathy in mice [168]. In transformed retinal Müller (glial) cells in hyperglycemia-like conditions, RVS decreased acetylation of histone H3 and could thus prevent the inflammatory changes that contribute to various ocular diseases such as DR [169]. Further studies are needed to examine whether RSV could modulate epigenetic alterations in ocular diseases.

## 10. New RSV Formulations and Clinical Trials

### 10.1. New Formulations of RSV

Several formulations have been developed to efficiently deliver RSV in the eye, including nanoparticle complexation and lipid-based encapsulation. For instance, RSV-loaded polyethylene glycols (PEGs) modified chitosan (CS) nanoparticles (NPs) by an ionic gelation method that was developed and tested both in vitro and in vivo in a rabbit model of glaucoma [170,171]. Data showed that this formulation improved corneal permeation compared with RSV dispersion, and that NPs can evoke an initial burst of RSV release of 45%, followed by a controlled release [165]. In vivo, the formulation was shown to efficiently reach the cornea and retinal choroid, and to significantly reduce IOP by 4.3 ± 0.5 mm Hg for up to 8 h in normotensive rabbits [170,171]. In a cellular model of AMD (ARPE-19 cells), it was also demonstrated that the use of RSV-loaded poly(lactic-co-glyolic acid) (PLGA) nanoparticles (optimized formulation with a particle size of 102.7 nm) improved RSV cellular uptake and increased RSV-induced inhibition of VEGF expression [172]. Although not tested as yet in vivo, this RSV formulation, either alone or in combination with conventional anti-VEGF therapies, may be promising in reducing VEGF-induced neovascularization in an AMD context. In epithelial D407 cells, Ruginǎ et al. [173] used a microencapsulated RSV formulation based on porous CaCO_3_ templates and polyelectrolyte layers covered by rhodamine 6G (Rh6G). Their so-called as-designed PMs-Rv-Rh6G microcapsules were internalized by D407 cells grown in normal and high glucose-induced inflammation conditions (to mimic DR), and reached the cell nucleus within 24 h. This formulation bioavailability was correlated with the inhibition of the expression and secretion of VEGF as assessed by ELISA. Additionally, the use of RSV loaded into lipid-cyclodextrin-based nanoparticles also seems to increase RSV bioavailability and to potentiate RSV-induced ROS scavenging capacities compared to RSV alone [174]. Besides nanoparticles or delivery vehicles, the design of an RSV prodrug formulation has also emerged. For instance, Valdés-Sánchez et al. [175] developed an RSV prodrug, 3,4′-diglucosyl resveratrol (JC19), and tested its efficiency in an experimental mouse model (autosomal recessive RP.Rd10 mice). They injected JC19 subretinally on postnatal day 13, and showed, 15 days post-injection, that JC19 significantly delayed the loss of rod photoreceptors associated with the maintenance of rhodopsin expression and the preservation of their electrical responses to light stimuli. Mechanistically, the authors suggest that Sirt1 activation by JC19 could explain the beneficial effects of the formulation.

### 10.2. RSV and Clinical Trials

Over the past decade, preclinical and clinical trials (Table 2) have shown that RSV could have a beneficial effect on health. Indeed, clinical trial has revealed that RSV supplementation in normal and healthy subjects can have antioxidant and anti-inflammatory properties in response to a high-fat, high-carbohydrate meal [176], which supports the fact that RSV could be used clinically in humans without toxicity.

Preclinical studies have shown that RSV has beneficial effects in ocular diseases, particularly AMD, glaucoma, cataract, DR, and vitreoretinopathy. Indeed, in vivo and in vitro studies have shown the anti-angiogenic potential of RSV in AMD [71] and DR [106]. One randomized and double-blinded clinical trial including 72 patients showed that supplementation with *trans*-resveratrol can lower VEGF levels in peritoneal effluent [177], thereby supporting the notion that RSV could be effective at reducing neovascularization in patients. Another clinical trial performed in the USA in octogenarians also showed that oral administration of Longevinex^®^, which is a combination of RSV with quercetin, ferulic acid together with vitamin D3 and a cooper/iron/calcium binding molecule called IP6 (inositol hexaphosphate), could improve retinal structure and visual function [178] as well as reduce neovascularization [179]. Moreover, a clinical trial on 19 type 2 diabetic patients supplemented with RSV revealed that it can not only be used to reduce oxidative stress and activate the AKT pathway, but also to improve insulin sensitivity [91]. Indeed, preclinical trials have shown that RSV can restore insulin levels in vitro.

### 10.3. RSV and Association with Other Therapeutics

Much evidence has been provided with regard to the bioactivities of RSV alone, both in vitro and in vivo. Nevertheless, RSV could also be useful in combination with conventional therapies. Indeed, in a cancer context, it was shown that RSV can act as a therapeutic adjuvant and is able to potentiate the efficacy of chemotherapies. Some studies have also shown the adjuvant potential of RSV in ocular diseases. For instance, in an experimental model of glaucoma, treatment with RSV either alone or in combination with riluzole significantly delayed RGC loss [181]. Very interestingly, Subramani et al. showed that RSV was able to reverse the adverse effects of bevacizumab on cultured ARPE-19 cells [182]. Indeed, numerous patients treated with anti-VEGF therapies show clinical complications due to repeated intravitreal injections [183,184]. In the presence of RSV, Notch signaling gets activated. This activation, along with dephosphorylation of Erk 1/2 and MEK, has been shown to be a major driver of the functional restoration of RPE cells treated with a RSV/bevacizumab combination compared to those treated with bevacizumab alone [182].

## 11. Safety of RSV

Concerning the toxicity of RSV, various studies have shown that RSV does not exhibit cytotoxicity in animal models and normal cells at the concentrations usually used in vitro (up to 100 µM) or in vivo (up to 500 mg/kg/day; see Table III) [185,186,187,188]. Moreover, some clinical studies have shown that RSV is safe in humans at various doses. Indeed, after daily RSV administration (0.5, 1.0, 2.5, or 5.0 g) for 29 days in 40 healthy volunteers, no significant adverse effects were observed, only mild to moderate gastrointestinal symptoms at 2.5 and 5.0 g RSV [189]. Other reports on trials of RSV in humans after single [190,191] or multiple daily doses of up to 600 mg/d administered over 2 or 3 days [192,193] show that RSV is safe under the tested conditions. Recent clinical studies have shown that patients with confirmed colorectal cancer present high levels of RSV metabolites, especially RSV-3-*O*-sulphate, RSV-3-*O*-glucuronide, and RSV-4-*O*-glucuronide, accumulated in the colorectum, and we have recently shown that these metabolites have anticarcinogenic properties. Thus, this poses the question as to whether RSV or RSV metabolites could be accumulated in eyes and ocular tissues and whether it is the aglycone molecule or its metabolites that are active. In fact, Wang et al. measured *trans*-RSV and its main metabolites in human eyes in patients with rhegmatogenous retinal detachment that were orally supplemented with Longevinex^®^ (containing 100 mg of *trans*-RSV; one capsule daily for a total of three doses prior to surgical tissue resection) [194]. HPLC/MS-MS was performed to quantify *trans*-RSV, RSV-3-*O*-sulfate, RSV-3-*O*-glucuronide, and RSV-4′-O-glucuronide on tissue samples. The investigators showed that RSV and its main metabolites were detectable in conjunctiva, aqueous humor, and vitreous humor (i.e., 17.19 ± 15.32 nmol/g RSV in conjunctiva and 62.95 ± 41.97 nmol/L RSV-3-*O*-sulfate in aqueous humor). These data suggest that RSV and its main metabolites can be found in human eyes after supplementation and could potentially participate at the tissue level in the treatment of ocular diseases.

## 12. Conclusions

There is compelling evidence that RSV can act on various pathologies in vivo such as coronary heart damage, cancer, and degenerative disease, by affecting various pathways. Along these pathways, RSV is able to act on common targets such as reactive oxygen species, lipids mediators, apoptosis, pro-inflammatory mediators, and angiogenesis. Through these mechanisms, RSV could prevent age-related ocular diseases (i.e., AMD, glaucoma, cataract, and DR) and could protect eyes against environmental factors (such as diabetes, hypertension, stress, UV light, acrolein found in cigarette smoke, and air pollution).

Finally, through its pleiotropic properties/mechanisms of action, RSV could constitute a good candidate to prevent ocular diseases and may provide a novel strategy to enhance the efficacy of therapy currently used. More preclinical studies are required to provide further insights into RSV’s potential adjuvant activity.

## 13. Methods

For this review, a systematic search of PubMed (https://pubmed.ncbi.nlm.nih.gov/) was conducted to identify studies on RSV in ocular cells, experimental animals, or humans in relation to ocular diseases up to December 2020. The search term “resveratrol” was used in combination with “ocular diseases,” “eye diseases,” “retinal cells,” “oxidative stress,” “reactive oxygen species” (ROS), “age-related macular degeneration,” “AMD,” “glaucoma,” “cataract,” “diabetic retinopathy,” “corneal infection,” “vitreoretinopathy,” “epigenetic,” and “clinical trials.” The search was limited to English-language studies.

## Figures and Tables

**Table 1:** *Trans*-resveratrol (*Trans*-RSV) concentrations in various food sources.

Sources	*Trans*-RSV (µg/g)	References
Hop	0.5	[33]
Peanuts	5.1	[34]
Peanut butter	0.3	[34]
Grape skin	27.5	[35]
Kojo-Kon	523	[34]
Blueberries	0.03	[36]

**Table 2:** Clinical trials with resveratrol with ocular measurements.

Author	Year	Region	Study Design	No. of Participants per Group Age (Mean ± SD)	Dose/Frequency/Duration/Follow-Up	Effects	Ref
Lin, C.T. et al.	2016	China	Randomized double-blinded trial	*n* = 72	(150 or 450 mg/d) *trans*-RSV or placebo during 12-week treatment Visits were scheduled at 0, 4, 8, and 12 weeks after treatment	Appearance rates of VEGF, Flk-1, and Ang-2 were more significantly reduced in the high-dose group versus the placebo group, but not in the low-dose group.	[177]
Wang S. et al.	2016	China	Randomized and divided into two groups matched by age and gender	34 participants randomly divided into two groups by age and gender matched, 11 women and seven men with a mean age of 25.44 ± 1.46 years (age range, 23–29 years) for the study group; eight women and eight men aged between 23 and 28 years with a mean age of 24.88 ± 1.26 for the control group.	Longenivex (100 mg of *trans*-RSV per capsule) against placebo All OCT scans were performed at the same time of day (between 9:00 a.m. and 12:00 p.m.).	A statistical increase in choroidal thickness (by EDI-OCT) 1 h after Longevinex ingestion compared with baseline measurements.	[180]
Richer S. et al.	2014	USA		*n* = 3 (two males and one female): Case 1: 64 y/o Caucasian with suspected glaucoma and photophobia, atrophic AMD, and diabetes with declining visual function in the right eye, had been on L/RV for 2.5 years and was maintaining visual function; Case 2: 89 y/o Caucasian with chronic kidney disease and cataracts, had been on L/RV for 3 years maintaining his visual function requirements to retain his driver’s license; Case 3: 67 y/o Caucasian with bilateral polypoidal choroidal vasculopathy (PCV), a treatment–resistant AMD variant, worse in the right eye. Improved retinal/choroid structure was observed.	Cases 1, 2: 2, 5 years Case 3: 2 years with Longevinex containing 100 mg of RSV)	Broad bilateral improvements in retina and choroid structure and function, visual acuity, contrast sensitivity, and glare recovery over a long time period, contrary to what might be expected due to aging and the natural progression of the patient’s pathophysiology. No side effects were observed.	[179]
Richer S. et al.	2013	USA		*n* = 3 (two males and one female): Case 1: 86 y/o morbidly obese male and advanced AMD; Case 2: 88 y/o female with bilateral wet AMD; Case 3: 75 y/o male with diabetes and dry AMD who developed wet AMD.	100 mg micronized/micro-encapsulated trans-RSV in Longevinex formulations.	Case 1 showed a Snellen visual acuity improvement by seven lines at 6 weeks and better IR choroidal circulatory images during the same period. Case 2 showed bilateral improvement in visual function and near resolution of retinal fluid after 2 weeks. Case 3 reported better vision in 5 days with L/RV, and objective retinal and visual restoration similar to anti-VEGF therapy wasobserved after 52 days of treatment	[178]
